# Prosthetic joint infections and legal disputes: a threat to the future of prosthetic orthopedics

**DOI:** 10.1186/s10195-021-00607-6

**Published:** 2021-11-09

**Authors:** Giuseppe Basile, Mario Gallina, Alberto Passeri, Rosa Maria Gaudio, Nicolò Castelnuovo, Pasquale Ferrante, Giorgio Maria Calori

**Affiliations:** 1 Trauma Surgery IRCCS Orthopaedic Institute Galeazzi, Piazza Tricolore 2, 20129 Milano, Italy; 2 Monselice, Italy; 3 Department of Morphology, Surgery and Experimental Medicine, University of Ferrara, Ferrara, Italy; 4 Città Studi Clinical Institute, Milan, Italy; 5 Department of Biomedical, Surgical and Dental Sciences, University of Milan, Milan, Italy; 6 Department of Reconstructive and Prothesic Revision-Surgery and Sepsis, San Gaudenzio Clinic-High Speciality Institute, Novara, Italy

**Keywords:** Orthopedic prosthetic surgery, Infections, Risk management, Malpractice, Medico-legal problems

## Abstract

Prosthetic joint infections (PJI) represent one of the major problems in orthopedic prosthetic surgery. The incidence of PJIs varies according to the site of intervention, and different published case studies report occurrence at 0.5 to 3.0% in the event of first implants, with a significant greater risk in the case of prosthesis revisions. The diagnosis of prosthetic infections is seldom simple, needing a multi-specialist approach, which includes the accurate collection of patient anamnesis, its clinical evaluation, the evaluation of inflammation biomarkers, and the use of imaging techniques. It is essential to identify the bacteria responsible for the infection not only for an accurate diagnosis, but also to select the correct antibiotic treatment. Failure to identify the bacteria involved makes it impossible to establish targeted systemic antibiotic therapy. In developed countries such as Italy, the right to health is guaranteed by the Constitution, where the institutions that provide health services must be staffed by a team of medical professionals that can guarantee the safest possible health pathways. Risk management represents the set of actions aimed at improving the quality of the care provided, the adherence to guidelines and good care practices with the final objective of guaranteeing patients’ safety. All hospitals, including the ones where prosthetic orthopedic surgery is performed, must adopt clinical risk management procedures which, through prospective tools aimed at preventing errors and complications and by retrospective methods, permit the identification of critical points in the different phases of the process and propose actions for improvement. The constant increase in litigation for malpractice in Western countries, especially in Italy, calls for special attention to the problem of PJIs and the in-depth assessment of medico-legal problems, also considering the new legislative initiatives in the field of medical malpractice. Hospitals need to tackle the onset of PJIs in a transparent and linear fashion by constantly informing the patient on their progress.

## Highlights

- Prosthetic joint infections (PJI) represent one of the major problems in orthopedic prosthetic surgery.
- The diagnosis of prosthetic infections requires a multispecialist approach, from accurate anamnestic collection, to clinical evaluation and biomarkers of inflammation, to the use of imaging techniques.
- The identification of the bacteria responsible for the infection is essential for the timely diagnostic setting and the correct antibiotic treatment.
- Risk management procedures, adherence to guidelines and good care practices, to prevent errors and complications.

## Introduction

In recent decades, the evolution and changes in the demographic composition of the population in developed countries and the concurrent progress of surgical technologies and materials used for prostheses in surgical interventions have led to a significant increase in the number of hip and other types of prostheses implanted on a global scale. A consequence of the increase of prosthesis implants has been the increase of a small but important number of different types of complications, including defects of positioning, defects in the material of the prosthesis and, most importantly, the possibility of prosthetic joint infections (PJI) [1, 2].

The onset of microbial infections after prosthetic interventions can cause serious clinical problems with negative consequences for patients, resulting, in the most serious cases, in surgery to remove the prosthesis itself, an occurrence that is becoming more and more often the subject of legal disputes that see patients filing charges against surgeons and hospitals for malpractice [3, 4].

Some aspects of prosthetic joint infections will be examined in this brief review of the scientific literature. We will detail epidemiological aspects, the different presentations of PJIs, their associated risk factors, their microbiological aspects, the importance of risk interventions management, and finally the medico-legal aspects. This last will focus on legal disputes against doctors and healthcare facilities by patients who develop these infections, with particular attention to what happens in Italy.

## Epidemiology of PJI

PJIs occur, according to the most recent data from the literature, in 0.5 to 3% of all implanted prostheses and up to 20% in the case of prosthesis revisions. However, from an epidemiological point of view, PJIs are very relevant because the frequency of prosthetic implants continues to increase in all developed countries of the world [5]. In the majority of cases, patients undergoing this type of surgery are often elderly, who frequently suffer from other pathologies. Patients who develop a periprosthetic infection usually undergo several surgeries, sometimes with exacerbation of the infectious pathology, leading to the explant and replacement of the prosthesis and patient dissatisfaction. This often results in a medical-legal dispute where the patient requests compensation from the orthopedic surgeon and/or the hospital where the surgery was performed [6, 7].

The development of PJIs is a serious complication associated with increased patient morbidity and has high health and social costs. One and a half million hip prostheses are implanted in the world every year. Italy is one of the European countries where the greatest number of hip replacement surgeries are performed. Every year, out of about 700,000 surgeries in Europe, over 100,000 are performed in Italy: only surpassed by Germany and France. It is important to emphasize that the number of implants increases by about 5% every year.

Joint prosthetic interventions on subjects who are under 65 and even 50 years of age have also become quite frequent in Europe and in Italy.

## Risk factors associated with the development of PJI

PJIs are the undesired result of the complex interaction of several factors according to the type of microorganism involved, the type of implanted prosthesis, and the characteristics of the patient undergoing surgery. In this short report we will focus on patients’ characteristics and the type of microorganism involved, with particular emphasis on the ensuing therapeutic needs.

Different bacteria use different mechanisms to colonize prosthetic material, and biofilm is one of the most efficient of these mechanisms. *Staphylococcus epidermidis* can produce a glycocalyx, or a polysaccharide with adhesive properties, which adheres to the prosthetic material and allows the bacteria to replicate without being attacked by the immune system or by antibiotics. *Staphylococcus aureus*, on the other hand, requires host tissues (collagen and fibronectin in particular) to carry out its pathogenic activity in prosthetic infections. The sequence of mechanisms that determines the adhesion of Gram-negative pathogens to the joint prosthesis is currently not known in detail. But we know that the type of prosthetic material used, as well as the regularity of its surface, influences bacterial adhesion.

### Patient’s related risk factors

Like many other surgical procedures, the implantation of knee, hip, or shoulder joint prostheses can result in bacterial infections to the prosthesis itself, and possibly to the adjacent bone tissues. Because the number of prosthesis implants is continuously increasing, especially due to the increase in the age of the population in developed countries, and to the increase in traumatism, numerous epidemiological studies are available to help define the risk of prosthetic infection [8, 9].

First, the risk of knee prosthesis infection is relatively low, ranging from 0.5 to 3%, depending on a series of factors mainly related to the subject undergoing the surgery and, to a lesser extent, the surgical technologies and setting. The following patient-related factors that can increase the risk of prosthetic infection, as indicated by *Workgroup 1: Mitigation and Education*, of the International Consensus on Periprosthetic Joint Infection, Philadelphia (2013), need to be mentioned:

- Presence of systemic or local infection elsewhere;
- Previous surgery in the same location;
- Decompensated diabetes malnutrition;
- Obesity (BMI > 30);
- Alcohol abuse;
- Drug addiction;
- Chronic renal failure;
- Chronic hepatitis B or C, HIV;
- Immunosuppression;
- Previous prolonged hospitalization or hospitalization in a nursing home.

PJIs are the leading cause of failure in knee prostheses, are the third most likely cause in hip prostheses in the United States, [10] and are a key factor in Europe. PJIs can occur with acute hip replacement following a fracture [11]. When approaching such a complex pathology as PJIs, decisions must be based on the objective evaluation of all risk factors. To support the best outcome, it is therefore critical to define the condition of the “patient at risk” by examining and addressing all comorbidities and fragilities [12].

The Patient at Risk Scoring System (PARSS), similar to the Non Union Scoring System (NUSS) [13, 14] already developed in traumatology, represents a new scoring system to assist surgeons when choosing the correct treatment in PJI surgery, following an “algorithm of treatment.”

### Intraoperative risk factors

Intraoperative risk factors include the use of large prostheses; the duration of the surgical intervention (> 3 h); the formation of a hematoma from a surgical wound; incorrect skin incisions with excessive surgical dissection; possible inadequacy of the operating room (quality of the air, disinfection of the environment, compliance with regulatory standards); suitable sterilization; accurate disinfection of operators’ hands; use of suitable gloves, gowns and drapes; accurate disinfection of the operating field; antibiotic prophylaxis and local antibiotic prophylaxis; limited “traffic” in the operating room; compliance with check-lists; and hand-off protocols.

### Postoperative risk factors

Postoperative risk factors are ascribable both to the hospitalization phase (sterile dressings, clinical and instrumental monitoring) and to the post-hospitalization phase, and mainly concern the dissemination of the infection from other organs or skin ulcerations.

Respiratory, urinary, and dental infections can trigger transient bacteremia, with potential risks of prosthetic infection. This risk is higher in the first 2 years after surgery.

Transferring surgical patients to post-surgical care facilities or to the care of territorial medical-nursing services has resulted in a reduction of the hospitalization period and to an increase in home care. It is therefore necessary to expand the concept of hospital infections to infections related to health and socio-health care. A care-related infection (ICA) is thus defined when it appears in a patient when cared for in a hospital or other healthcare facility and was not present and not in incubation at the time of the patient’s admission.

## The different types of PJI

The classification of PJIs in relation to onset time has been the subject of wide debate. According to a relatively simple classification that originates from numerous scientific reports, they are divided into three types: early (intraoperative), delayed (intermediate), and late.

### Early PJI (EPI)

The infections that occur within the first month after surgery are defined as early: these, in most cases, are due to intraoperative contamination or arise in the immediate period after surgery.

Generally, early infections originate from or begin as surgical wound infections and are characterized by a striking clinical picture. The pathogens involved are mainly *S. aureus*, usually oxacillin-resistant (MRSA), coagulase-negative staphylococci (CONS) and several aerobic Gram-negative bacteria.

### Delayed or intermediate PJI (DPI)

In these types of infections, pain and suppuration arise either when the surgical wound has already healed or more than a month after surgery. Culture examination reveals that intermediate infections are mainly caused by the skin’s commensals and low virulence coagulase-negative staphylococci: this explains the lapse in time required to produce the symptomatic infection. A share of delayed infections, those that occur between the 6th and 12th month, arrive from hematogenous spread from other locations, and the spectrum of the pathogens involved is wider. Differentiating between delayed and late PJI is not easy, and in some cases delayed manifestations can be considered late.

### Late onset PJI (LCI)

The infections of the prosthesis that occur more than 1 year after surgery are defined as late (LCI) and are mostly due to hematogenous contamination originating from outbreaks elsewhere in the body or from skin lesions, dental abscesses, or urinary infections. These LCIs are characterized by a more subtle onset and a difficult clinical interpretation. LCIs are also more frequently polymicrobial, because hematogenous dissemination facilitates simultaneous colonization of low virulence pathogens already present in situ.

A much more detailed classification of PJIs has been proposed by Tsukayama [15]. This scheme divides PJIs into four categories, partly based on the presumed mode of infection and the time elapsed since surgery, adding acute hematogenous infections (AHI) to the three types described above.

## Diagnosis of PJI

One of the major problems clinicians face when dealing with PJIs is the fact that the diagnosis is often difficult and not promptly defined, especially when the attending physician does not pay sufficient attention to the few and not always specific symptoms which may be indicative of a PJI.

According to a traditional view of clinical studies on PJIs, they are associated with signs and symptoms such as local redness, heat, and swelling, or stiffness and pain in the joint with the appearance of continuous drainage from the wound or fistula. However, the analysis of a large number of cases and data from the literature has shown that these clinical signs of high diagnostic value, which are also relatively easy to identify, are not always present. Therefore, it is always necessary to suspect a PJI in the presence of pain in the joint prosthesis combined with one or more of the risk factors previously listed.

In a more modern view, the diagnosis of a prosthetic infection is based on clinical examination, blood chemistry, elevated serum erythrocyte sedimentation rate (ESR) and serum C-reactive protein (CRP) concentration, microbiological investigations on the wound exudate, investigations of blood cultures and, if deemed possible, investigation of joint needle aspiration fluid and imaging techniques such as X-ray and CT scan [16, 17].

The symptomatology of a prosthetic infection is mainly characterized by local redness, heat, swelling, pain, and stiffness, while the appearance of secretions from the wound or draining fistula can be completely absent or, in some cases, appear later.

In the diagnostic field, various instrumental and laboratory tests are available, none of which, however, has a 100% sensitivity and specificity. In joint prosthesis infections, laboratory microbiological diagnostics can make use of culture tests performed on samples taken directly from the bone outbreak, or from superficial samples, even if several studies have shown a low concordance between the results obtained with the two methods [18].

In patients with prosthetic joint infection, culture examination of the aspirate of the synovial fluid is characterized by a sensitivity of 82–94% and a specificity of 94–97%, depending on the cases.

A Gram stain of the synovial fluid has a high specificity (> 97%) but a low sensitivity (< 26%). Periprosthetic tissue cultures are more likely to identify the pathogen responsible for the infection. At least three samples must be tested to increase the sensitivity of the exam.

Imaging techniques include conventional radiology, CT, MRI, and nuclear medicine. Highly sensitive and specific PETs and SPECTs represent additional diagnostic aids. Because no single test can provide diagnostic accuracy by itself, it is necessary to combine laboratory, microbiological, histopathological, and instrumental investigations to correctly classify prosthetic osteoarticular infections.

## Microbiological etiopathogenesis of PJIs

In recent years the microbiology of PJIs has undergone numerous changes which are worth discussing for the impact they have had on the prevention, diagnosis, and treatment of these infections. Several factors have contributed together or singly to these changes. Worth mentioning are the changes in the population of patients undergoing orthopedic prosthetic surgery, the constant evolution of microorganisms both from a genomic and pathogenetic point of view, and the introduction and innovation of diagnostic methods for microbiological infections.

One of the main changes in the characteristics of patients who undergo surgery, including PJ surgery, is their age which, especially in developed countries, continues to increase, with a consequent increase in pre-, peri-, and postoperative complications.

The number of older patients undergoing surgery is increasing due to changing demographics, surgical and anesthetic technical advances, and new additional healthcare expectations. Older patients not only exhibit acute or chronic problems requiring surgery but have concurrent multimorbidity and physical decline, which often includes immune system impairment and other common geriatric syndromes. These additional issues increase the risk of adverse postoperative outcomes, especially postoperative medical and functional complications including infection of the surgical site [19].

Like all living things, bacteria have the evolutionary potential to modify their genome and their replicative characteristics. Continuous evolution and the consequent genesis of variants determines the development of new abilities. Among the most important in microbiology, and in medicine in general, is the possibility for bacteria to become resistant to antibiotics.

All bacteria are able to generate antibiotic-resistant variants, but this happens only with the intervention of external factors in their environment. According to the European Center for Disease Prevention and Control (ECDC) there are at least three scenarios that can lead to antibiotic resistance.

The first is the completely inappropriate use of antibiotics in the treatment of viral infections. The second is the prescription of antibiotics when a precise etiological diagnosis has not yet been made in the presence of an infection. This happens if a broad-spectrum antibiotic is prescribed when the microorganism responsible for the infection is not known, often resulting in the elimination of many bacteria except for the one responsible for the infection. The third scenario is the misuse, in terms of duration, dosage, and/or frequency of antibiotic treatments, both during hospitalization and, more frequently, in non-hospitalized patients.

In this regard, it is worth remembering that penicillin, at the time of its discovery and its introduction in the treatment of infections, was paradoxically effective against many bacteria due to the fact that no antibiotic had ever been used before.

It can be stated with certainty that over time we have gone progressively from a phase in which the bacteria responsible for these infections were easily attacked by antibiotics, to the current situation in which, with a few exceptions, the majority of PJIs are caused by bacteria resistant to some antibiotics and even multi-drug-resistant bacteria (MDRO).

Antibiotic-resistant bacteria, which are the most frequent etiological agents of PJI, are briefly listed below.

The bacteria most frequently involved in joint prosthesis infections are *Staphylococcus aureus* oxacillin-resistant (ORSA), methicillin-resistant negative coagulase staphylococci such as oxacillin-resistant *Staphylococcus epidermidis* (ORSE) and methicillin-resistant *Staphylococcus hominis*, vancocin resistant enterococci (VRE) such as *Enterococcus faecium*, *Pseudomonas aeruginosa,* and multi-resistant *Acinetobacter baumannii*. The resistant organism isolated most often in joint prosthesis infections is without doubt ORSA.

Over the past decade, the number of infections caused by ORSA has increased significantly, both in many European countries and in the United States. This increase is partly associated with the development of numerous healthcare facilities, such as hospitals, nursing homes, dialysis centers, long-term care centers, and communities, in which antibiotics are often excessively and improperly prescribed and used, favoring the selection and spreading of this germ. The excessive use of cephalosporins and fluoroquinolones has been associated with the selection of resistant methicillin strains [20].

Established risk factors for the development of ORSA infections [21] are: hospitalization or stays in nursing homes and clinics, contact with patients with MRSA, being over 70 years of age, being male, hospitalization in an intensive care unit, and previous use of antibiotics (especially if wide spectrum).

Coagulase negative staphylococci are frequently implicated in joint prosthesis infections. Knee and hip prosthesis infections caused by coagulase negative staphylococci, especially *S.* *epidermidis* and *S.* *hominis*, are often nosocomial. Coagulase negative staphylococci may have a lower sensitivity to common antibiotics and antimicrobial agents than *Staphylococcus aureus*. In addition, most coagulase negative staphylococci are resistant to beta lactams; *Staphylococcus haemolyticus* can also be resistant to vancomycin. There have been reports of osteoarticular infections from methicillin-resistant coagulase-negative staphylococci, especially MRSE, which can be very difficult to treat [22].

In recent years we have seen a selection of vancomycin-resistant enterococci (VRE). Immunodeficiency, taking broad-spectrum antibiotics, multiple surgeries, prolonged hospitalizations, previous use of vancomycin, chronic renal failure, malignancies, and organ transplants, are the most important risk factors for the development of VRE hospital infections [23].* Enterococcus faecium* represents the most frequently resistant enterococcus to vancomycin [24].

*Pseudomonas aeruginosa* and *Acinetobacter baumannii* are aerobic Gram-negative bacteria that are becoming a frequent cause of hospital infections, as in the case of nosocomial pneumonia associated with a high mortality rate [25]. These organisms are mainly responsible for septicemias, infections related to the use of central venous catheters, intestinal and urinary tract infections, pneumonia, endocarditis, and meningitis, but they can also cause osteomyelitis, septic arthritis, periprosthetic infections, and infections of the skin and soft tissues (for example surgical wound infections).

These organisms are intrinsically resistant to many antibiotics due to the structural characteristics of their outer membrane. In addition, they can rapidly develop additional resistance mechanisms during antibiotic treatment, causing problems in the treatment of serious infections.

*P. aeruginosa* and *A. baumannii* are ubiquitous germs due to their ability to grow and multiply in very adverse chemical-physical conditions. *P. aeruginosa* can grow in distilled water: contaminated taps represent an important reservoir of infection for sensitive patients in hospitals and healthcare facilities [26].

Recent studies have given a significant boost to the awareness of the microbiological aspects of PJI by better defining the association of different bacteria with the different types of PJI described earlier, highlighting the importance of some bacteria and other microorganisms that, until a few years ago, were not associated with PJI.

A significant contribution from this point of view was a very extensive study conducted in Spain which included a large number of PJI patients. This work highlights that early PJIs (EPIs) are significantly associated with antibiotic-resistant bacteria such as MRSA, VREs, and MDROs, while AHI are associated with less virulent bacteria. A very interesting fact that emerged from the same work is that the bacterial species involved in PJI change over time after surgery, with a decrease of the most well-known and known pathogens and an increase of CONS which are often difficult to typify [27].

Taken together, these data reaffirm the need for empirical broad-spectrum antibiotic therapy while the results of cultural investigations are not yet available.

With reference to microorganisms that had a previously unknown role in PJI, we must focus on the growing importance of mycobacteria, including tuberculosis mycobacteria (MOTT) [28].

Mycobacteria in PJI are now isolated with a certain frequency directly at the site of infection with the aid of new gene amplification techniques such as polymerase chain reaction (PCR) which have enormously increased the sensitivity and speed of diagnostic methods for mycobacterial infection [29, 30].

Finally, a fungal etiology of PJIs is a very rare event which must, nonetheless, be taken into consideration when other microorganisms cannot be isolated. Recent data seem to indicate a slight increase in these types of infections, in particular *Candida* species [31].

## PJI therapy

When a prosthesis becomes infected, regardless of the etiology and the clinical pathway, the therapy to reclaim the prosthesis and eradicate the infection is difficult and complex since it can only be based on antibiotic treatment and, when antibiotic treatment fails, a series of different or consecutive surgical interventions may be necessary.

In short, the treatment of prosthetic PJI can consist only of antibiotic therapy which, although targeted, does not always guarantee success in eradicating the infection. Debridement can also be performed in association with antibiotic therapy; it can be effective in a high percentage of cases, even over 80% if performed promptly following the initial surgery. In case of failure, it is necessary to remove and replace the prosthesis; an operation which can be carried out in a single procedure if the microorganism involved has been isolated, in which case a new cemented prosthesis with a specific targeted antibiotic, is replanted. In more complex cases, it will be necessary to remove the prosthesis and implant an antibiotic spacer, cemented and filled with targeted antibiotics. A new prosthesis, generally not cemented, must be implanted after 8–12 weeks from the explant. In the postoperative period, systemic antibiotic therapy, if possible, should be continued for at least 6–8 weeks, first intravenously, then orally. The various classifications proposed constitute a guide for the choice of treatment, and Tsukayama’s work, which distinguishes acute, delayed, and late prosthetic infections, still remains valid.

Antimicrobial therapy combined with surgery is needed to treat PJIs [32]. A common management approach is to start broad-spectrum antimicrobial agents after obtaining intraoperative samples for culture [33]. The importance of adequate initial empirical antimicrobial therapy in the outcome of infections is well known and appears to be critical in patients with PJI treated with debridement, antibiotics, and implant retention [34]. Vancomycin combined with a broad-spectrum beta-lactam, such as piperacillin-tazobactam, has recently been studied as an initial treatment, but has been associated with a high rate of adverse effects. After the pathogen has been identified and the results of antimicrobial susceptibility are available, the most effective narrow-spectrum antibiotic regimen is selected for continued therapy. However, a significant number of patients (5–35%) have negative cultures [15]. In this situation, empirical antimicrobial therapy is even more important, but more difficult to select, considering that the patient will have to undergo treatment for the several weeks or months that are needed to treat a PJI.

## The role of risk management in facing PJI

Clinical risk management in healthcare represents the set of actions implemented to improve the quality of healthcare services and ensure patient safety.

In this case, the risk considered is related to a potential loss linked to an adverse event; the operational aspects (“management”) therefore concern the hypothesized relationship between risk and possible loss.

Clinical risk management is defined as an “organized supervision aimed at identifying, assessing and reducing where necessary, risks for patients, visitors and healthcare personnel in general,” [32] in order to improve the quality and safety of services and at the same time prevent economic losses.

Therefore, the role of the risk manager is to identify the main risk areas, analyze potential critical issues, and identify corrective actions, the actual effectiveness of which must be monitored over time [35]. Healthcare safety is the result of an integrated and coordinated strategy between health professionals from different backgrounds that focuses on the patients and the outcome of the activities aimed at them [36, 37]. The task of the clinical risk manager must be understood as an articulated set of strategies and actions to address the main risks in the healthcare setting, such as hospital-acquired infections [38, 39].

The prosthetic infection should not be considered an incident between the doctor and the patient, but its occurrence concerns the whole process involving the place of care, its organization, its processes and the protocols applied, the doctor and all specialists involved, and all health professionals and other users who interact with the place of care [40]. This complete view of the process allows us to understand the immense variability of factors that can contribute to the onset of hospital infections and, among these prosthetic infections, where the insertion of artificial materials exponentially increases these occurrences [40, 41]. Considering the large number of factors at play and the complexity of management, the role of the risk manager is to coordinate and supervise the treatment process, focusing on safety and re-evaluating the various steps of care processes with the trained eye of a risk assessment professional [42].

The collaboration between professionals, and in particular the infectious disease specialist, is fundamental. They must be involved not only at the onset of the prosthetic infection but at the beginning of the process, by collaborating in the drafting of protocols concerning the disinfection of environments and patients, the choice of skin disinfectants, and antibiotic therapy: selecting the drugs and administration times based on the organizational needs of the facility.

In this regard, please note that any guideline used in a healthcare facility must be adapted to the organizational requirements of that specific facility. This step must also be reviewed together with the risk manager because it could generate critical issues [42]. An operative instruction or a therapeutic protocol applied to a different institution could lead to different results in terms of incidence of infections.

Hospital infection control committees with microbiological monitoring, case analysis, and data collection [41–43] are among the tools a health facility must implement in the area of clinical risk management. Regional operational guidelines, which list the main strategies to be followed each year, include the reporting of nosocomial infections of sentinel cases to the competent bodies.

PJIs are a significant part of nosocomial infections and, as such, represent one of the main elements to be considered in the management of clinical risk inside hospitals, including those with orthopedic operating units in which joint prostheses are implanted. Progress achieved in the fields of microbiology, bacteriology, immunology, and the introduction of antibiotics to the market in the 1940s has contributed to spreading the illusion that infections can be definitively eradicated. But, in reality, infections continued to represent an important “complication” in hospital environments, and their trend, in the absence of control programs, has been proven to increase over the years. Currently, nosocomial infections are one of the health emergencies on a global scale, due to incidence and mortality, with heavy repercussions on the health system, both for the use of those resources necessary for their management and for the consequences on the health of the population [44].

Furthermore, the progressive introduction of new healthcare technologies in hospital care has created the conditions for new complex interactions between pathogenic microorganisms and the biomaterials used. In particular, the implantation of foreign bodies in humans increases the susceptibility of surgical wounds to infection: in surgeries with insertion of prostheses the incidence of infection is always higher compared with similar surgeries without the implantation of prostheses [37].

Unfortunately, the most interesting data, considering the implications of damage to the reputation of the health system, the ensuing loss of patients’ trust, and probable legal disputes, come from the indicators linked to claims for damages.

The analysis of claims in Italy has numerous limitations since data collection is not homogeneous and does not allow an accurate analysis. The most complete data come from the mapping of the claims from the region of Lombardy’s public health institutions. Claims for damages in Lombardy, in northern Italy, are distributed according to a fluctuating trend, which has shown a decrease in recent years.

Over 20 years, the hospital specialties that have generated the most requests for civil damages are: orthopedics and traumatology (15.4% of cases), ERs (12.3%), and general surgery (9.3%). The most frequent cases in orthopedics and traumatology concern surgical errors (54.0%), diagnostic errors (10.4%), infections (9.3%), and therapeutic errors (8.8%) [35]. In general, the last few years have seen a progressive reduction of therapeutic errors, surgical, and diagnostic errors, but an increase in cases of infection which include surgical site infections, prosthetic materials, and sepsis [32].

Recently the Italian Parliament has issued a new law that regulates possible litigation between patients, doctors, and hospitals. The Italian State Law No. 24 of 8 March 2017 states that safety of care is a constitutive part of the right to health [45]. Access to safe care is sanctioned as a constitutional right with an intrinsic commitment by the institutions that provide services to the person to be staffed with a team that can guarantee the safest possible treatment pathways.

The healthcare facility must appoint a clinical risk manager who, using prospective tools aimed at preventing errors and complications and by retrospective methods, identifies the critical points in the various stages of the process and proposes improvement actions. In fact, the pathophysiological complexity and a correct approach are constantly and rapidly evolving and require frequent clinical and organizational updates, to be adapted to the specific institution.

Analyzing the process by collecting data and measuring them through spontaneous reports from operators, reporting adverse events from the complaints office, reviewing medical records, analyzing adverse events, clinical auditing, updating of existing procedures, creating quality indicators, pharmacovigilance tracking systems, and monitoring of antibiotic use are among the activities aimed at managing hospital infections. Furthermore, participation in global research, surveillance, and prevention programs, such as the monitoring of sentinel microorganisms through a regional and national reporting system, is mandatory to ensure an adequate comparison with standards. In the organizational system of the hospital, the role of the risk manager is strategic in spreading the so-called risk culture among health professionals at all levels, with a non-guilty approach aimed at learning from mistakes to improve the quality and safety of care. In this context, coordination between the risk manager and the legal and administrative figures that manage the compensation risk becomes essential. To this end, a claims assessment committee usually operates in Italian hospitals. The committee includes a health director, a risk manager, a legal team and a physician specialized in forensic medicine. Once the case has been examined, if critical issues have been identified in the conduct or in the organization of the health facility and the conditions for compensation due to damages to health have been confirmed, the risk manager must identify any existing issues to implement suitable corrective actions to prevent the recurrence of similar events. Therefore, the claims assessment committees, adequately structured and organized for the assessment and management of claims, are to be seen as the link between clinical practice and the legal sphere, to exchange opinions and evaluate the legal guidelines that will outline the application of new laws and rulings issued in the health sector from different points of view.

To reduce litigation, especially in nosocomial infections, it is also necessary to involve the patients and their families and to consider them critical agents for safer healthcare. In fact, by addressing all the people involved, such as citizens, family members, and volunteers, we contribute to the quality of care. Handouts containing information and the measures to be adopted by patients for their safety and wellbeing must be distributed to increase health and awareness. The patient thus becomes an active party in contributing to making healthcare safer.

## Legal considerations for PJI according to Italian rules

Our brief review of the complex etiopathogenetic aspects of nosocomial PJIs highlights the difficulty of establishing a direct causal link between the onset of the infection and the culpable behavior of the orthopedic surgeon. In an environment where multiple factors overlap, this can be a cause of aggravation.

In Italy, civil liability provides for compensation for damages from an unlawful act. Law 24/2017 sanctioned the distinction, indeed already contemplated by the Civil Code, between contractual liability (ex art. 1218 of the Civil Code), which pertains to the hospitalization structure, public or private, and to the healthcare system that carries out its work in the private sector, and non-contractual liability (pursuant to art. 2043 of the Civil Code), which is based on the principle of “neminem laedere” and rests on the healthcare worker employed by the institution. In essence, the contract is actualized in the institution, because it is the contracting party that is obliged to perform at a certain standard and who is responsible for its execution, through its employees.

In this case, in accordance to art. 2236 of the civil code, the patient must prove only that he has suffered damage and the causal relationship between it and the medical intervention, while the doctor must prove that he acted correctly and that the case did not constitute a special difficulty. This view was confirmed by the Court of Legitimacy (e.g., Cass. 19/5/1999, n. 4852; Cass. 4/2/1998, n. 1127; Cass. 16/2/2001, n. 2335) until decisions made by the United Civil Sections n. 13533/2001 and n. 577/2008 which deal with the issue of the burden of proof in the event of non-fulfillment. In this case, the principle has been definitively established that whoever acts in court must only prove the negotiation or legal source of his right, limiting himself to an allegation against the counterparty, based on the circumstances. If this burden is fulfilled, the jurisprudence unequivocally believes that it is up to the defendant to prove that it has complied with the obligation by implementing, with diligence, skill, and prudence, the means available to modern medicine. If such proof is not provided, guilt is presumed.

In fact it is almost impossible, when the nature and nosocomial provenance of the bacterium that infected the hospitalized patient has been established with certainty, and also when it is certain that the infection did not occur before hospitalization from the relative septic pathology, to identify the place in the hospital where the infection occurred, the precise moment, the behavior of health professionals, technicians, or auxiliaries, or other possible ways in which the infection might have been spread, such as the lack of or insufficient cleaning/sanitizing procedures (now often contracted to external firms) [46].

This lack of knowledge means that the only certainty is that the infection is of a hospital nature, and therefore liability cannot be established because the deficiency on the part of the hospital cannot be definitively proven.

It is therefore necessary to ask whether hospital infections, in particular periprosthetic infections, are not only foreseeable but also preventable, or whether they cannot be completely avoided even in the presence of satisfactory, concrete, and effective measures implemented by the hospital concerning the sanitation of the premises, personnel, tools, or any other possible source of contact and contamination, as well as the implementation of appropriate protocols.

Scientific studies and the most up-to-date statistics show that preventability is a desirable but unreachable goal. In general, it can be said that a large part of the reduction in the number of infections recorded in recent years is the result of shorter hospitalization stays and implementation of the rules introduced for correct blood utilization and the implementation of risk management practices [40].

There remains a significant share of infections, in particular periprosthetic infections, that invariably occur in all hospital facilities. For these, effective strategies must be identified and diversified on the basis of hospital wards, the site of infection, the type of patient, the type of procedure, and also in terms of sustainability and viability.

It must therefore be highlighted that there is absolutely no a priori preventable infection: all of them are theoretically preventable, but each must be assessed collectively in a legal setting by the medical examiner and the infectious disease specialist (Law 24/2017, article 15, paragraph 1) in relation to the specific situation, circumstances, and related acts.

If the respondent party must submit evidence, often circumstantial, that the harmful event (infection with a nosocomial microorganism) was possible/foreseeable, but in the specific case was not preventable (falling within the cases that medical science has listed as events that can also escape the correct implementation of the protocols provided for, and adopted by the healthcare facility), the only way to fulfill this evidentiary burden is to provide positive proof that all the specific scientific measures so far devised to avoid, or at least reduce to the minimum, the risk of infections have been implemented. If this evidence is provided, liability should be excluded, because one must accept that portion of risk intrinsically related to the medical-surgical activity, that is, of minimum risk or tolerable risk, according to a principle that can be borrowed from other sectors, as prescribed by Legislative Decree 81 / 2008, which refers to the UNI EN ISO 12100 directives (citation of DL 81/2008).

Other elements of assessment can be inferred from the adequacy of prophylaxis and antibiotic therapy, the fact that health professionals have followed guidelines and no unnecessary, harmful, or inadequate treatments have been carried out.

At this point we can agree with Javad Parvizi and Thorsten Gehrke, who in an interview [28] thus concluded the discussion on the topic: “The only effective and ultimately the only correct recommendation is to openly address the complication of PJI. The patient should be informed of the possibility of an infection as soon as possible and undergo appropriate diagnostic tests. This can only be done through an open and honest dialogue with the patient. Complaints are unnecessary and irrelevant, due to hygiene standards observed in most operating rooms around the world. As a rule, PJIs should be considered a random event. Responsibilities can be attributed to the surgeon or to the attending physician only in the case of delays or a wait-and-see approach to diagnostic outcomes and subsequent treatment. In a nutshell, the best thing is to be honest”.

## Conclusions

Infections in prosthetic surgery are increasing due to the staggering increase in arthroplasty procedures. This determines a growing incidence of medico-legal disputes, which represent a serious problem of interpretation from a clinical, diagnostic, and therapeutic point of view. The evaluation of each case must be performed starting from sound scientific premises, in accordance with reference guidelines produced by reference scientific institutions. After a thorough examination, each patient must be rigorously classified, taking into account all comorbidities, i.e., all risk factors associated with his/her medical history.

There is no doubt that only a rigorous analysis of the medical history of each patient, considered as a whole individual, will permit the establishment of the actual degree of fragility and the increased risk, specifying and justifying which treatment, in accordance with a specific algorithm, is adequate and suited to treat any serious consequences. This innovative approach will most likely prove to be useful for an objective, and therefore comparable, critical analysis.

## Data Availability

Not applicable.

## Abbreviation

PJI: Prosthetic joint infections
